# Preclinical assessments of safety and tumorigenicity of very high doses of allogeneic human umbilical cord mesenchymal stem cells

**DOI:** 10.1007/s11626-024-00852-z

**Published:** 2024-02-29

**Authors:** Sze-Piaw Chin, Nik Syazana Saffery, Kong-Yong Then, Soon-Keng Cheong

**Affiliations:** 1 Cytopeutics Sdn Bhd, Bio-X Centre, Persiaran Cyberpoint Selatan, Suite 2-3, 2nd Floor, Cyber 8, 63000 Cyberjaya, Selangor Malaysia; 2 CMH Specialist Hospital, Jalan Tun Dr. Ismail, 70200 Seremban, Negeri Sembilan Malaysia; 3 M. Kandiah Faculty of Medicine and Health Sciences, Universiti Tunku Abdul Rahman (UTAR), Bandar Sungai Long, 43000 Kajang, Selangor Malaysia; 4 Cryocord Sdn Bhd, Cyber 8, 63000 Cyberjaya, Selangor Malaysia

**Keywords:** UC-MSC, Safety, Toxicity, Tumorigenicity, Inflammation

## Abstract

Human umbilical cord-mesenchymal stem cells (hUC-MSCs) have been widely investigated as a new therapeutic agent to treat injuries and inflammatory-mediated and autoimmune diseases. Previous studies have reported on the safety of low-dose infusion of hUC-MSCs, but information on the cell behaviour at higher doses and frequency of injection of the cells remains uncertain. The aim of the present study was to demonstrate the safety and efficacy of hUC-MSCs by Cytopeutics® (Selangor, Malaysia) from low to an extremely high dose in different monitoring periods in healthy BALB/c mice as well as assessing the tumorigenicity of the cells in B-NDG SCID immunocompromised mice. Umbilical cord from two healthy human newborns was obtained and the isolation of the hUC-MSCs was performed based on previous established method. Assessment of the cells at different doses of single or multiple administrations was performed on healthy BALB/c mice in dose range finding, sub-acute (7 d and 28 d) and sub-chronic periods (90 d). Tumorigenicity potential of Cytopeutics® hUC-MSCs was also evaluated on B-NDG immunocompromised mice for 26 wk. Single or multiple administrations of Cytopeutics® hUC-MSCs up to 40 × 10^6^ cells per kilogramme of body weight (kg BW) were found to have no adverse effect in terms of clinical symptoms, haematology and other laboratory parameters, and histology examination in healthy BALB/c mice. hUC-MSCs were also found to reduce pro-inflammatory cytokines (IL-6 and TNF-α) in a dose-dependent manner. No sign of tumor formation was observed in B-NDG mice in the 26-wk tumorigenicity assessment. Single or multiple administration of allogenic Cytopeutics® hUC-MSCs was safe even at very high doses, is non-tumorigenic and did not cause adverse effects in mice throughout the evaluation periods. In addition, Cytopeutics® hUC-MSCs exhibited immunomodulatory effect in a dose-dependent manner.

## Introduction

Human umbilical cord mesenchymal stem cells (hUC-MSCs) have certain advantages such as abundant sources, ease of extraction and low immunogenicity (Xie *et al* 2020; Xu *et al *2022). hUC-MSCs possess greater proliferation and slower senescence activity after an extensive culture period. This ultimate characteristic of hUC-MSCs is crucial to ensure sufficient cell population is obtained for wide-scale clinical use (Baksh *et al* 2007; Jin *et al* 2013). Furthermore, hUC-MSCs do not only possess greater multipotency than other adult stem cells, but also apparently do not form tumors when transplanted in the body (Park *et al* 2016). We have previously demonstrated the safety of Cytopeutics® allogenic hUC-MSCs infusion in healthy volunteers and its immunomodulatory and anti-inflammatory effects in a dose-dependent manner up to 2 × 10^6^ cells/kg BW (Chin *et al* 2020).

Preclinical studies on animals are important to obtain information on the safety, efficacy and toxicity of any therapeutic products, prior to their application in clinical trials. Several animal toxicology studies have been conducted to evaluate the efficacy and safety of MSCs from predominantly bone marrow sources (Rengasamy *et al* 2016; Tayebi *et al* 2022). Safety, toxicity and tumorigenicity studies looking at hUC-MSCs were more limited (Wang *et al* 2012; Kannaiyan *et al* 2017; Chan *et al *2022; Xu *et al* 2022; Pan *et al* 2023). Chan and co-workers reported that hUC-MSCs infused at a dose of 10 × 10^6^ cells/kg BW intravenously on Sprague Dawley rats were considered safe and did not alter the haematological and biochemical as well as histological parameters in animals within the 12-wk study (Chan *et al* 2022). Another study examining intravenous (IV) injection of placenta-derived MSCs at the dose of 10 × 10^6^ cells/kg BW also did not cause fatality or unusual clinical manifestations in Wistar rats in a 14-d observation study (Subramani *et al* 2022). There is limited data on safety and efficacy of hUC-MSCs at higher doses. Yet increasingly more recent studies have been using multiple dosing at short intervals or with much higher doses to maximise the reparative and immunomodulatory effects of MSCs. For example, in Graft-Versus-Host-Disease (GVHD) where there is severe immune-mediated injury and reactions, Murata *et al* (2021) demonstrated that it was beneficial for acute GVHD patients to receive 8 infusions of 2 × 10^6^ cells/kg BW MSCs each time within a 4-wk period. Hess *et al* (2017) explored the effect of using hUC-MSCs of up to 1.2 × 10^9^ cells/subject in patients with acute ischemic stroke which approximates to 17 × 10^6^ cells/kg BW (assuming human body weight is 70 kg). In line with the current understanding on MSCs to treat GVHD, we are currently embarking on a clinical trial that aims to evaluate the efficacy and safety of providing up to 3 infusions of allogeneic hUC-MSCs infusions at 5 × 10^6^ cells/kg BW in patients with grades II–IV acute GVHD (NCT03847844).

The present animal study is aimed at demonstrating the safety and toxicity limits of hUC-MSCs at different single doses and/or in multiple infusions using 2 × 10^6^ cells/kg BW, 5 × 10^6^ cells/kg BW up to 15 × 10^6^ cells/kg BW, 20 × 10^6^ cells/kg BW, 40 × 10^6^ cells/kg and 100 × 10^6^ cells/kg BW in healthy BALB/c mice model. The tumorigenicity risk of the cells was also evaluated on immunocompromised B-NDG mice in the same GLP-compliance facility. The study also aimed to compare the immunomodulatory effect of Cytopeutics® hUC-MSCs at different doses. This will provide further insights into the efficacy and safety profiles of higher and multiple doses of hUC-MSCs, specifically to support the use of multiple doses of 2 × 10^6^ cells/kg BW and 5 × 10^6^ cells/kg BW treatment regimes for future clinical studies in stroke, diabetes and acute GVHD.

## Methods

### Cell culture and production

The isolation and cell culture procedures were applied based on our previous work (Chin *et al *2020). After delivery of full-term and healthy babies, umbilical cord samples were obtained following written consent from both parents. The cell processing procedure was conducted in a certified Good Manufacturing Practice (GMP) laboratory in accordance with Malaysia Guidelines for Stem Cell Research and Therapy. The hUC-MSCs were isolated based on adherence to flask’s surface after the high-quality umbilical cords were digested. The expansion of the cells were done in established growth medium kept in 37 °C, 5% CO_2_ and 95% air incubator. Non-adherent cells were removed and replaced with new growth medium until it reached confluence on the next 3 d. The cells were then cultured in new flasks until the required cell number was achieved. The cells’ first early passages were cryopreserved for future applications. In the present study, cells were thawed and the expansion was done up until passage 6. The hUC-MSCs were then tested for quality control including immunophenotyping, differentiation assays and contaminants testing for bacterial, fungal and mycoplasma.

### Research and animal ethics

The study was carried out at Syngene International Ltd., (Bengaluru, India), in compliance with OECD Principles of Good Laboratory Practice (GLP). The study protocol was approved by the Institutional Animal Ethics Committee (IAEC Protocol No: SYNGENE/IAEC/1210/10–2020). All experimental animal procedures were done in accordance with the recommendations of Association for Assessment and Accreditation of Laboratory Animal Care International (AAALAC) and Regulations of Committee for the Purpose of Control and Supervision of Experiments on Animals (CPCSEA), Government of India.

### Animals

A total of 77 male and 77 female BALB/c mice (age, 10 to 11 wk old; weight, 19 to 25 g) for toxicity study, and a total of 24 male and 24 female B-NDG SCID mice (age, 64 to 68 d old; weight, 21 to 29 g) for tumorigenicity study were obtained from Envigo (Limburg, Netherlands). All mice were kept in sterilised polycarbonate cages in a controlled room at 19–24 °C with a 12-h light/dark cycle, relative humidity of 40–70% and free access to food and water. The mice were acclimatised for 6 d in the experimental room prior to the initiation of dosing and were observed for clinical signs once daily during the acclimatisation period.

### Study design and dose selection

The study was designed based on “ICH M3 (R2), Guidance on Nonclinical Safety Studies for the Conduct of Human Clinical Trials and Marketing Authorization for Pharmaceuticals, Current Step 4 version, dated 11 June 2009”. Acute, sub-acute and sub-chronic toxicity assessments were applied in the present study along with tumorigenicity assay. The study was divided into 4 parts and was conducted in sequential order: dose range finding (part I), followed by repeated dose sub-acute study (part II), 90-d tumorigenicity study (part IV). Slow bolus intravenous injections of the Cytopeutics® hUC-MSCs were performed on BALB/c mice in toxicity studies while the tumorigenicity assessment was conducted using subcutaneous injection on immunocompromised mice strain, B-NDG SCID.

In the dose range finding study (part I), the mice were randomly assigned and equally distributed to different groups based on the doses, ranging from 2 × 10^6^ cells/kg BW to 100 × 10^6^ cells/kg BW and then monitored for 14 d.

In the sub-acute studies (part II), the dose selection was based on our current proposed clinical trials specifically for acute ischemic stroke and acute graft-versus-host disease (aGVHD) where we plan to give up to 3 infusions of 2 × 10^6^ cells/kg BW over a 28-d period, and up to 3 infusions of 5 × 10^6^ cells/kg BW over a 7-d period, respectively.

In the sub-chronic toxicity study (part III), we selected two doses of the hUC-MSCs which are 15 × 10^6^ cells/kg BW and 40 × 10^6^ cells/kg BW, followed by 90-d of observation post-injection. The reason for choosing 15 × 10^6^ cells/kg BW is because that is the cumulative dose of 3 infusions of 5 × 10^6^ cells/kg BW over a 7-d period that we intended for the GVHD clinical trial. The second dose of 40 × 10^6^ cells/kg BW is chosen as it was determined to be the upper safety limit in a single dose, as demonstrated in part I (dose-range finding study).

In tumorigenicity study (part IV), we selected the same doses as for part III which are 15 × 10^6^ cells/kg BW and 40 × 10^6^ cells/kg BW via intravenous injection. Subcutaneous injection of DLD-1 cells (200 × 10^6^ cells/kg) was applied on mice to induce tumor development as a positive control in the study. All mice injected with hUC-MSCs were observed for 180 d. However, the mice injected with DLD-1 tumor cells were euthanised on day-32 as the tumor size already reached 10% of their body weight.

**Figure 1.**
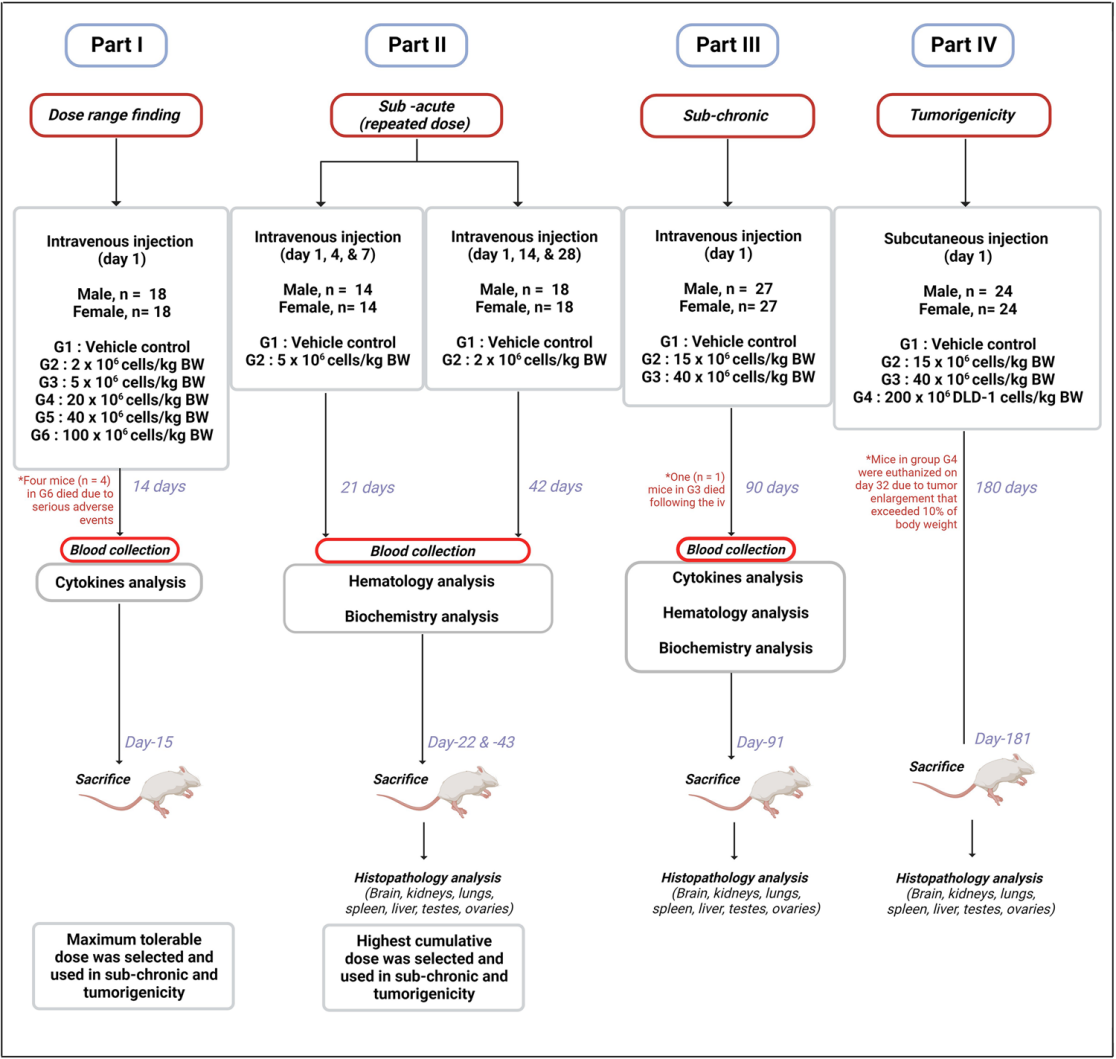
The flowchart of the toxicity and tumorigenicity studies of Cytopeutics® hUC-MSCs in mice model. The study was divided into 4 parts and was conducted in a sequential order; dose range finding (part I), followed by sub-acute repeated dose (part II), 90-d sub-chronic study (part III), and 26 wk tumorigenicity study (part IV). Image was created using Biorender.com.

Blood collection was performed via retro-orbital sinus on all surviving mice and processed according to the respective tests including cytokines; tumor necrosis factor (TNF-α), interleukin-6 (IL-6), interleukin-10 (IL-10), Creactive protein (CRP), haematology evaluation; red blood cell (RBC), haemoglobin (HGB), platelet (PLT), reticulocytes (RETIC), white blood cell (WBC), neutrophil (NEUT), lymphocytes (LYMPH), monocytes (MONO), eosinophils (EOS), basophils (BASO), and biochemistry evaluation; glucose (GLUC), blood urea nitrogen (BUN),urea (UREA), creatinine (CRE), alanine aminotransferase (ALT), aspartate aminotransferase (AST), alkaline phosphatase (ALP), gamma-glutamyl transpeptidase (GGTP), total protein (TP), sodium (Na), and potassium (K). A flowchart detailing the toxicity and tumorigenicity assays of the Cytopeutics® hUC-MSCs on mice models is simplified in Fig. 1.

### Single dose range finding assessment in BALB/c mice (14-d)

The first part of the study is the dose range finding (DRF) assessment of the Cytopeutics® hUC-MSCs which was conducted to evaluate the potential of systemic toxicity of the cells in BALB/c mice at a maximum tolerable dose (MTD) and a minimum lethal dose (MLD). In this study, Cytopeutics® hUC-MSCs were administered through single IV slow bolus injection at different dose levels: 2 × 10^6^ cells/kg BW, 5 × 10^6^ cells/kg BW, 20 × 10^6^ cells/kg BW, 40 × 10^6^ cells/kg BW and 100 × 10^6^ cells/kg BW or vehicle. The starting dose level at 2 × 10^6^ cells/kg BW was based on the proposed clinical dose for MSC-based treatments (Chin *et al* 2020, 2021; Law *et al* 2021). The dose at 5 × 10^6^ cells/kg BW was selected based on the findings and efficacy study of Cytopeutics® hUC-MSCs in the Phase I/II clinical trial GVHD study (NCT03847844). The other doses were set at 10, 20 and 50 times higher than the common clinical trial dose of 2 × 10^6^ cells/kg BW in order to establish the safety and toxicity limit. All 36 mice were observed daily for morbidity/mortality and clinical signs for 14 d. Detailed clinical examination was performed once weekly. Body weight and food consumption were recorded twice weekly.

### Sub-acute toxicity assessment for 7 d and 28 d with 14-d observation of Cytopeutics® hUC-MSCs in BALB/c mice

The second part of the study aimed to evaluate sub-acute toxicity, target organ toxicity and reversibility of any adverse effect of Cytopeutics® hUC-MSCs when administered thrice repeatedly over a 7- or 28-d period, followed by 14 d of observation in healthy BALB/c mice. In the 7-d repeated dose toxicity study, 14 male and 14 female BALB/c mice were randomly assigned to vehicle control group and treatment group (5 × 10^6^ cells/kg BW). The dose selection and the frequency of administration for 7-d repeated dose toxicity were based on our phase I/II GVHD clinical trial (NCT03847844). Treatments were administered as a single dose through IV bolus injection into healthy BALB/c mice on day-1, -4 and -7, followed by 14-d of observation. In the 28-d sub-acute toxicity study, 18 male and 18 female BALB/c mice were randomly assigned to vehicle control group and treatment group (2 × 10^6^ cells/kg BW) in which the administration frequency and dose were selected based on our phase II acute stroke clinical trial (NCT06129175). Treatments were administered as a single dose through IV bolus injection into healthy BALB/c mice on day-1, -14 and -28, followed by 14-d of observation. During the experimental period, all mice were observed daily for morbidity/mortality and clinical signs of toxicity, whereby body weight and food consumption were recorded twice weekly.

### Sub-chronic toxicity assessment (90-d) in BALB/c mice

The third part of the study investigated the 90-d sub-chronic toxicity of Cytopeutics® hUC-MSCs at 15 × 10^6^ cells/kg BW and 40 × 10^6^ cells/kg BW. The cells were administered once through single IV slow bolus injection into BALB/c mice, followed by 90 d of observation. 15 × 10^6^ cells/kg BW was chosen as it represented the highest cumulative clinical dose in the 7-d sub-acute repeated dose study, whereas 40 × 10^6^ cells/kg BW was chosen as it was the MTD in our acute toxicity study. A total of 27 male and 27 female BALB/c mice were randomly assigned to three groups receiving either vehicle control, 15 × 10^6^ cells/kg BW or 40 × 10^6^ cells/kg BW. All mice were observed daily for morbidity/mortality and clinical signs of toxicity. Food consumption was recorded once per week while water consumption was observed periodically on weeks 1, 7 and 13.

### Tumorigenicity assessment in B-NDG SCID mice

The tumorigenicity risk of hUC-MSCs was evaluated in the fourth part of the study, using immunocompromised B-NDG SCID mice (24 males and 24 females) at same two dose levels previously: 15 × 10^6^ cells/kg BW or 40 × 10^6^ cells/kg BW by single subcutaneous injection into the abdominal flank of mice. Colorectal adenocarcinoma (DLD-1) cells at a dose of 200 × 10^6^ cells/kg BW served as positive control, while normal saline served as vehicle control. We chose to administer the hUC-MSCs and the tumor cells subcutaneously so that the cells may remain much longer in the body (Leow *et al* 2015), as opposed to intravenous injection (Cao *et al* 2016). All mice were monitored periodically for 26 wk (181 d). Throughout the study, all animals were evaluated for morbidity/mortality, clinical signs observation, detailed clinical examination, tumor volume measurement, body weight and food consumption. All animals were sacrificed for a necropsy and gross pathology evaluation at the end of 26 wk or if the tumor volume measurement progression exceeded 2000 mm^3^.

### Haematology, biochemistry and cytokine analysis

Blood sample was collected at the end of observation days via retro-orbital sinus puncture under mild isoflurane anaesthesia. Although the blood collection was carried out on all surviving mice, the analysis varied among the studies. Blood samples collected from DRF and sub-chronic studies were subjected for cytokines (pro-inflammatory) evaluation using plasma, whereas whole blood analysis was performed on mice in the sub-acute and sub-chronic toxicity studies to observe the haematological and biochemistry status after Cytopeutics® hUC-MSCs treatment. Prior to blood collection, mice were fasted at least 3 h; however, water was provided ad libitum during the fasting period. Haematology assessment was performed using an automated haematology analyser (ADVIA 2120i, Siemens, Erlangen, Germany) whereas biochemistry parameters were estimated with a clinical chemistry system (Dimension Xpand Plus, Siemens). Meanwhile, plasma samples were outsourced to Syngene Biology, Bangalore, India for cytokine quantification using ELISA. The selected cytokines were including TNF-α, IL-6 and IL-10.

### Gross pathology examination and histopathology analysis

All surviving mice were euthanised by exsanguination under deep isoflurane anaesthesia at the end of the observation period of the study. Vital organs were harvested and weighed before complete necropsy and gross pathological examinations were performed on all animals. For histology analysis, tissues were preserved in 10% neutral buffered formalin. After fixation, the tissues were processed, embedded in paraffin, sectioned and stained with haematoxylin and eosin staining. The stained tissues were viewed under light microscope to evaluate the pathological changes. Peer review was performed by the in-house pathologist.

### Statistical analysis

Statistical analyses were performed using Pristima version 7.4.3 for toxicology studies in recording of mortality and morbidity, daily observations, detailed clinical observation, body weight gain, food consumption, haematology, biochemistry, organ weight, organ weight ratio and gross pathology. The data were analysed using Pristima built-in statistical tests. For comparative statistics, data were evaluated using Levene’s Test and ANOVA for homogeneity of variances, with significance at 5% level (*p* < *0.05*). For two group comparisons, data were subjected to Student *t*-test. Results are presented as mean ± standard deviation (mean ± S.D.) or mean ± standard error mean (mean ± SEM).

## Results

### Single DRF assessment (14-d) in BALB/c mice

**Figure 2.**
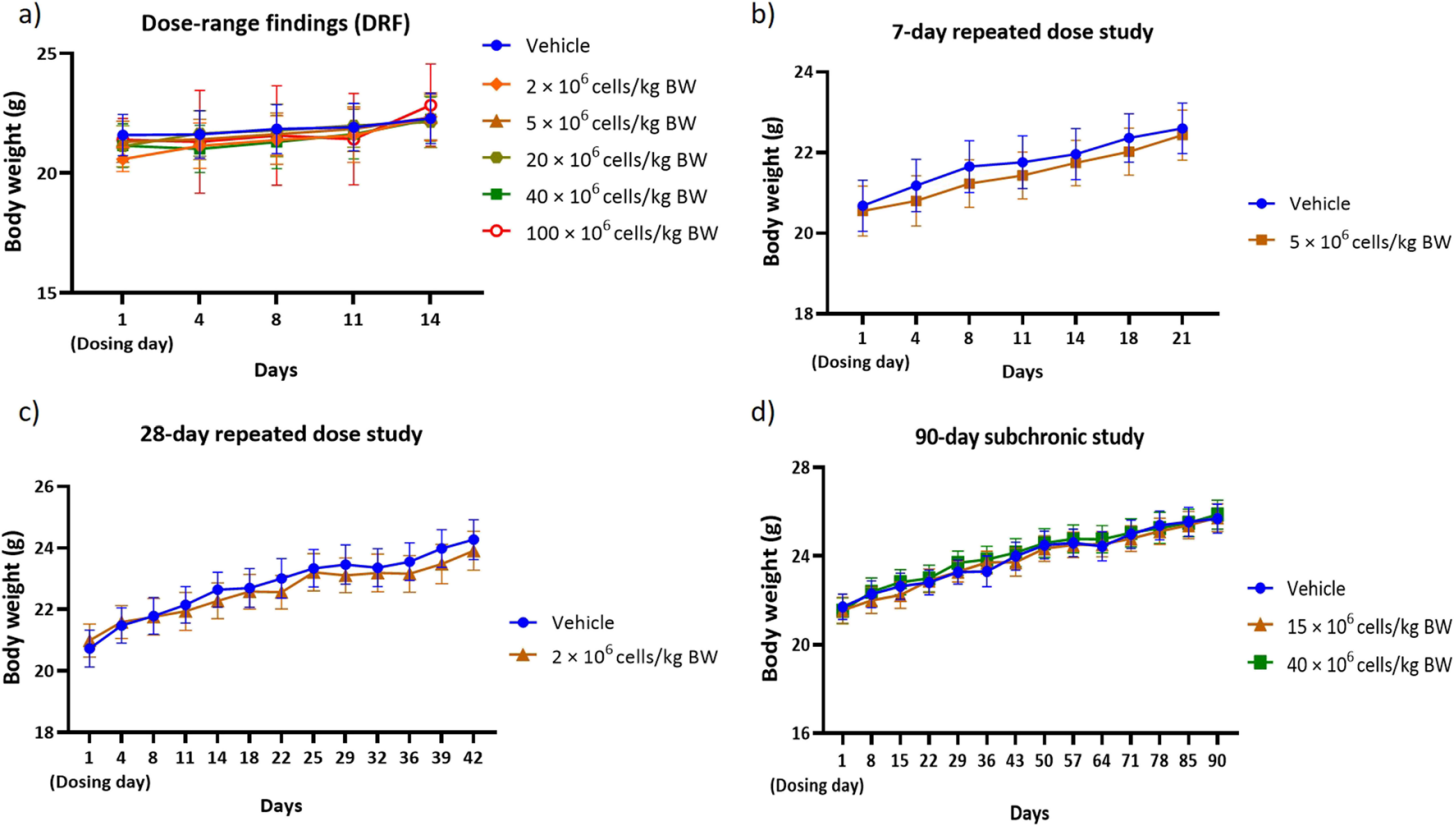
Summary of mice body weights (sex pooled) following IV administration of Cytopeutics® hUC-MSCs. Analysis of body weight from different toxicity studies including (*a*) DRF, (*b*) sub-acute (7-d repeated dose), (*c*) sub-acute (28-d repeated dose), and (*d*) sub-chronic (90-d). In general, consistent body weight gained was observed in all mice in the population. Data are presented as mean and the vertical bars represent standard error mean (SEM).

**Figure 3.**
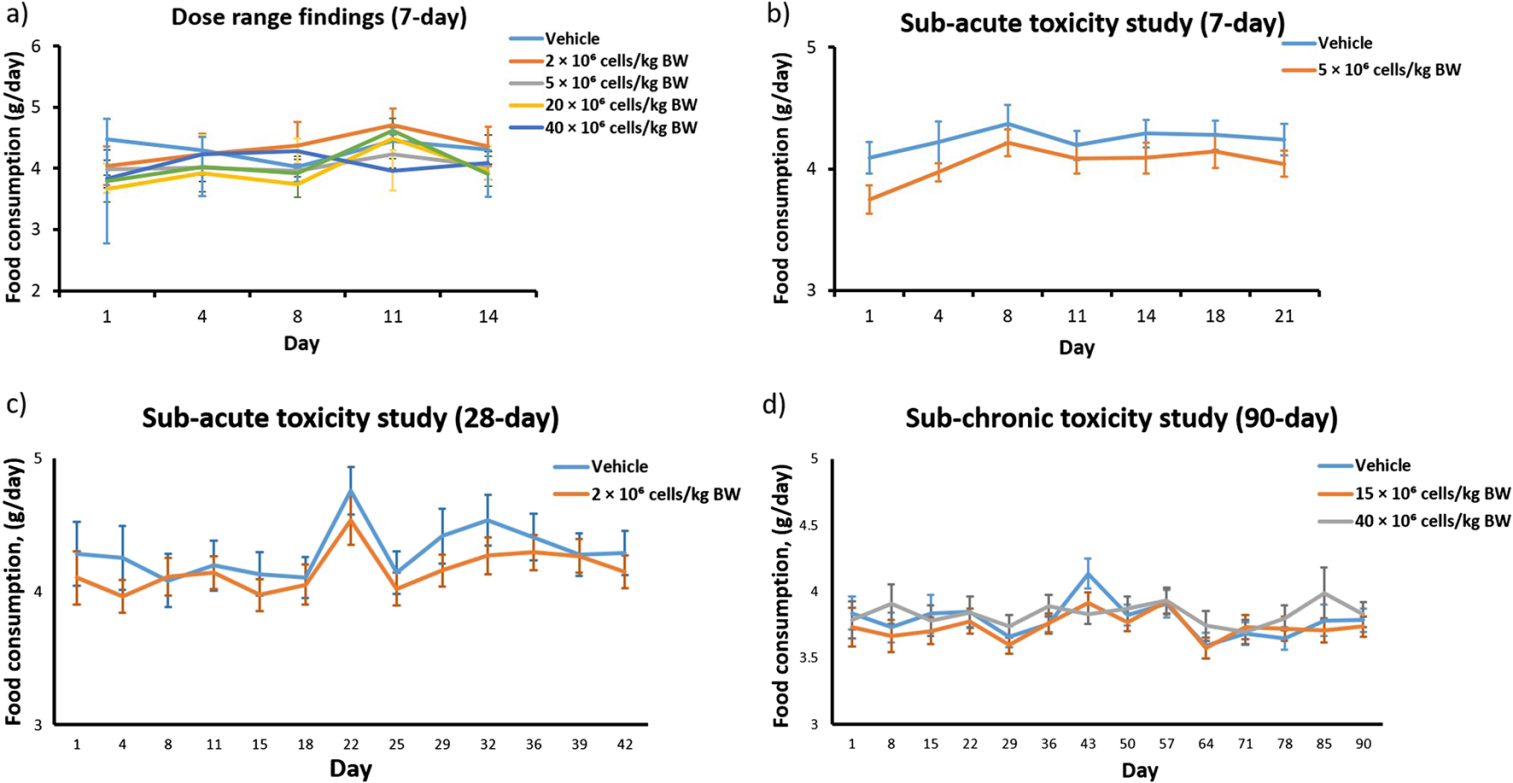
Summary of food consumption (sex pooled) following IV administration of Cytopeutics® hUC-MSCs. Analysis of food consumption from different toxicity studies including (*a*) DRF, (*b*) sub-acute (7-d repeated dose), (*c*) sub-acute (28-d repeated dose), and (*d*) sub-chronic (90-d) study. In general, food consumption of the mice in all toxicity studies seemed to be consistent. Data are presented as mean and the vertical bars represent standard error mean (SEM).

Based on our single DRF study, no abnormal clinical signs and mortality were observed in mice receiving Cytopeutics® hUC-MSCs of up to 40 × 10^6^ cells/kg BW. There were no significant changes in body weight (Fig. 2*a*) and food consumption (Fig. 3*a*) of the mice after the observation period. However, four out of six mice injected with 100 × 10^6^ cells/kg BW died (Fig. 2*a*). The animals that died showed clinical signs such as tremors and laboured breathing prior to death. During gross examination, white deposits in pericardium of heart and/or cornea of the eyes were noted in three out of four animals that died, while the fourth animal had no gross lesion during necropsy. Therefore, 40 × 10^6^ cells/kg BW was applied as the MTD and used for subsequent studies.

### Repeated dose in sub-acute toxicity assessment: 7 d and 28 d with 14-d observation of Cytopeutics® hUC-MSCs in BALB/c mice

Multiple IV infusions of the Cytopeutics® hUC-MSCs at 2 × 10^6^ cells/kg BW and 5 × 10^6^ cells/kg BW did not cause adverse effect in healthy BALB/c mice, demonstrating its safety at multiple IV administrations at these doses and intervals. Specifically, there were no deaths, abnormal clinical signs, body weight change (Fig. 2*b*, *c*) and food consumption (Fig. 3*b*, *c*) during the 14-d observation period.

### Sub-chronic toxicity assessment (90 d) in BALB/c mice

Similarly, we did not observe any adverse effect on body weight (Fig. 2*d*), food intake (Fig. 3*d*) and clinical parameters in BALB/c mice treated with single intravenous administration of Cytopeutics® hUC-MSCs at 15 × 10^6^ cells/kg BW. One male animal receiving 40 × 10^6^ cells/kg BW was found dead on day-1 of the dosing. Prior to death, clinical signs of severe deep respiration and convulsion (clonic) at post-dose were observed. The cause of death could not be determined due to the absence of gross or correlating microscopic lesions during necropsy, suggesting that the death was not caused by cell injection. The remaining mice from the same group showed mild clinical signs such as mild decreased activity, mild to severe deep respiration and clonic convulsions of duration 15 to 50 s in males and 15 to 20 s in females, on day-1 post-injection. Subsequently, all these mice recovered to normal from day-2 until terminal sacrifice.

### Haematological and biochemical evaluation following the IV administration of Cytopeutics® hUC-MSCs in BALB/c mice

Analysis of the blood for evaluation of haematological and biochemical parameters was performed as a standard procedure for toxicity study. Based on the findings, there were no significant changes in the haematological and biochemical parameters between the vehicle control and treated groups. Details of the measured parameters for the respective analysis are presented in Table 1 (haematological) and Table 2 (biochemical).

**Table 1.** Selected haematological parameters of sex pooled (male and female) BALB/c mice in toxicity studies of Cytopeutics® hUC-MSCs

Type of study	7-d repeated dose toxicity study		28-d repeated dose toxicity study		90-d sub-chronic toxicity study		
Group	Vehicle control	5 × 10^6^ cells/kg BW	Vehicle control	2 × 10^6^ cells/kg BW	Vehicle control	15 × 10^6^ cells/kg BW	40 × 10^6^ cells/kg BW
RBC (× 10^6 cells/μL)	10.31 ± 0.50	10.52 ± 0.31	10.3 ± 0.49	10.26 ± 0.41	10.77 ± 0.61	10.93 ± 0.52	10.55 ± 0.37
HGB (g/dL)	16.55 ± 0.50	16.78 ± 0.78	16.35 ± 0.44	16.33 ± 0.28	16.98 ± 0.69	16.82 ± 0.75	15.38 ± 2.78
HCT (%)	54.05 ± 1.97	54.53 ± 1.98	54.48 ± 2.40	54.68 ± 1.75	56.77 ± 2.87	56.80 ± 2.71	56.16 ± 1.99
MCV (fL)	52.48 ± 0.66	51.83 ± 0.5	52.9 ± 0.54	53.32 ± 0.56	52.73 ± 0.63	52.05 ± 1.04	53.22 ± 0.52
PLT (× 10^3^ cells/μL)	675.75 ± 38.19	665.75 ± 86.73	717.83 ± 65.09	762.18 ± 99.66	608.33 ± 178.18	603.00 ± 186.39	624.40 ± 76.06
RETIC (× 10^9^ cells/L)	341.58 ± 15.07	333.18 ± 10.27	293.23 ± 18.98	312.52 ± 41.11	305.37 ± 19.19	359.85 ± 85.76	313.44 ± 35.84
WBC (× 10^3^ cells/μL)	7.725 ± 1.67	6.97 ± 0.61	5.58 ± 1.11	5.08 ± 1.67	3.27 ± 0.77	3.39 ± 1.13	4.16 ± 0.63
NEUT (× 10^3^ cells/μL)	1.30 ± 0.37	1.05 ± 0.18	0.98 ± 0.21	1.17 ± 0.85	0.61 ± 0.26	0.83 ± 0.60	0.86 ± 0.11
LYMPH (× 10^3^ cells/μL)	6.12 ± 1.22	5.67 ± 0.57	4.35 ± 1.04	3.7 ± 0.95	2.34 ± 0.54	2.31 ± 0.58	3.02 ± 0.80
MONO (× 10^3^ cells/μL)	0.12 ± 0.05	0.10 ± 0.02	0.07 ± 0.02	0.07 ± 0.04	0.00 ± 0.00	0.00 ± 0.00	0.00 ± 0.00
EOS (× 10^3^ cells/μL)	0.16 ± 0.06	0.12 ± 0.04	0.14 ± 0.06	0.12 ± 0.04	0.26 ± 0.16	0.20 ± 0.17	0.21 ± 0.26
BASO (× 10^3^ cells/μL)	0.01 ± 0.01	0.01 ± 0.01	0.01 ± 0.01	0.01 ± 0.00	0.01 ± 0.01	0.01 ± 0.02	0.01 ± 0.01

Values are represented as mean ± SD, where *p* < 0.05 compared to vehicle control. Red blood count (RBC), mean corpuscular volume (MCV), white blood count (WBC), haematocrit (HCT), neutrophils (NEUT), haemoglobin (HGB), eosinophils (EOS), basophils (BASO), lymphocytes (LYMPH), monocytes (MONO), reticulocytes (RETIC), platelet count (PLT)

**Table 2.** Selected clinical chemistry parameters of sex pooled (male and female) BALB/c mice in toxicity studies of Cytopeutics® hUC-MSCs

Type of study	7-d repeated toxicity study		28-d repeated toxicity study		90-d sub-chronic toxicity study		
Group	Vehicle control	5 × 10^6^ cells/kg BW	Vehicle control	2 × 10^6^ cells/kg BW	Vehicle control	15 × 10^6^ cells/kg BW	40 × 10^6^ cells/kg BW
GLUC (mg/dL)	123.00 ± 7.05	129.24 ± 17.85	135.50 ± 18.81	134.66 ± 14.78	110.00 ± 19.80	101.43 ± 26.44	102.62 ± 22.77
BUN (mg/dL)	18.005 ± 3.54	19.268 ± 3.14	22.07 ± 4.37	23.45 ± 5.12	25.97 ± 9.33	26.05 ± 5.04	30.09 ± 10.63
UREA (mg/dL)	38.58 ± 7.59	41.28 ± 6.72	47.29 ± 9.37	50.25 ± 10.96	55.64 ± 20.00	55.83 ± 10.79	64.44 ± 22.76
CRE (mg/dL)	0.13 ± 0.03	0.147 ± 0.01	0.13 ± 0.06	0.14 ± 0.03	0.22 ± 0.08	0.19 ± 0.06	0.21 ± 0.06
ALT (U/L)	44.90 ± 13.95	42.44 ± 20.83	59.34 ± 36.63	37.1 ± 13.17	82.79 ± 40.81	80.71 ± 28.67	92.82 ± 46.03
AST (U/L)	89.26 ± 14.77	95.07 ± 9.17	88.77 ± 27.68	96.00 ± 29.04	124.33 ± 30.53	112.48 ± 22.79	124.63 ± 24.54
ALP (U/L)	190.52 ± 35.38	213.63 ± 18.29	186.43 ± 17.86	187.36 ± 25.40	134.19 ± 24.65	144.02 ± 30.24	154.00 ± 32.10
GGT (U/L)	5.48 ± 0.37	5.50 ± 0.09	6.47 ± 0.42	6.48 ± 0.77	7.21 ± 0.52	7.04 ± 0.60	7.14 ± 0.53
TP (g/dL)	3.85 ± 0.12	5.05 ± 0.15	4.96 ± 0.20	5.12 ± 0.18	5.20 ± 0.37	5.03 ± 0.56	5.30 ± 0.12
Na (mmol/L)	153.18 ± 0.67	153.77 ± 1.65	153.80 ± 1.68	153.87 ± 1.02	154.52 ± 3.45	155.58 ± 2.14	156.73 ± 2.19
K (mmol/L)	5.60 ± 2.53	5.44 ± 0.46	5.23 ± 0.39	5.30 ± 0.14	6.70 ± 0.80	6.15 ± 0.68	6.90 ± 1.17
CRP (ρg/mL)	2.90 ± 0.27*	3.33 ± 0.57*	2.90 ± 0.27*	3.87 ± 0.64*	3.69 ± 0.52	3.83 ± 0.70	3.52 ± 0.64

^*^Values were obtained from the DRF study (14 d) with single infusion of 2 × 10^6^ and 5 × 10^6^ cells/kg BW. Values are represented as mean ± SD, where *p* < 0.05 compared to vehicle control. Alanine aminotransferase (ALT), Aspartate aminotransferase (AST), Alkaline phosphatase (ALP), gamma-glutamyl transpeptidase (GGT), total protein (TP), creatinine (CRE), glucose (GLU), urea, blood urea nitrogen (BUN), potassium (K) and sodium (Na)

### Plasma inflammatory cytokine analysis in BALB/c mice in DRF and sub-chronic studies

**Figure 4.**
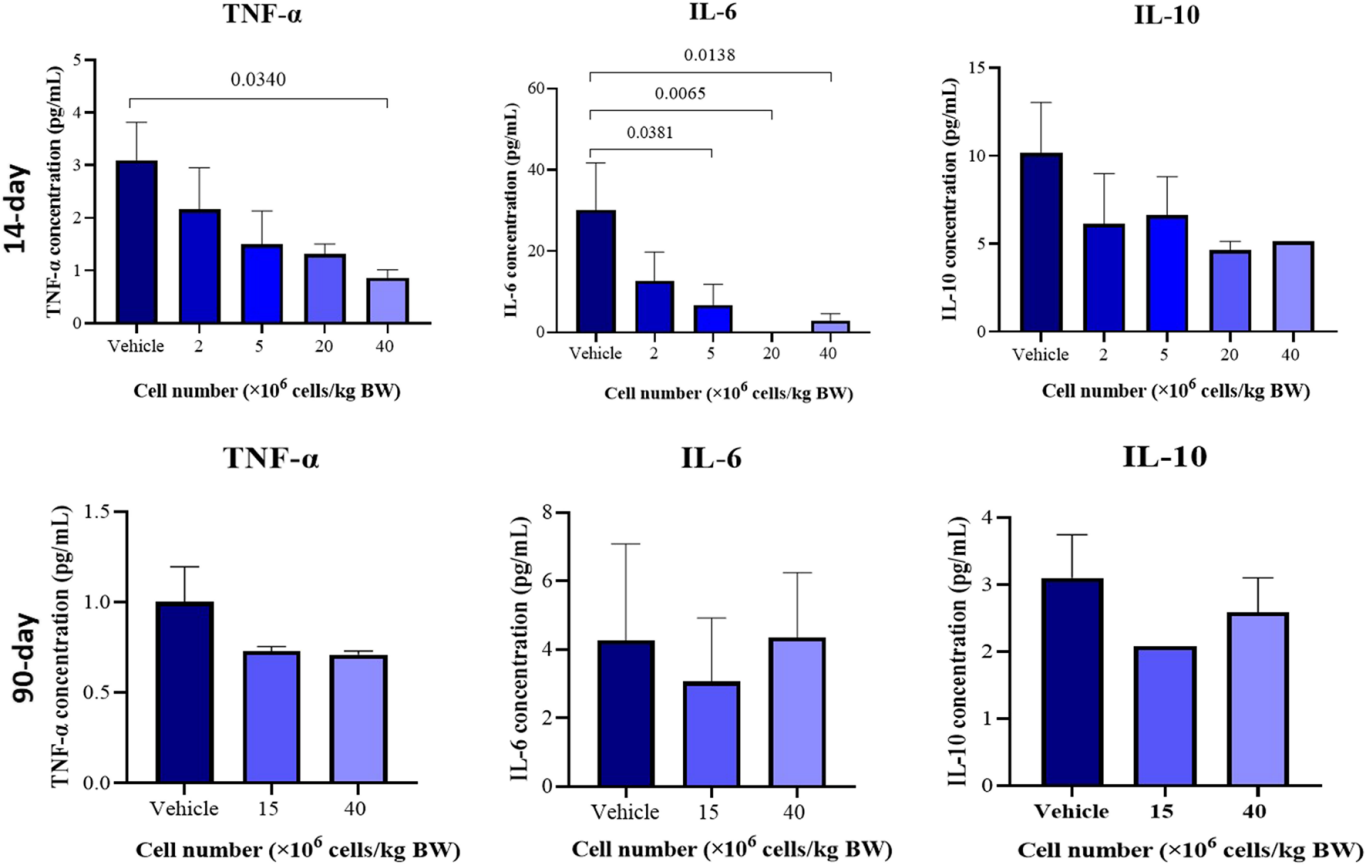
Cytokine levels in the plasma samples of mice (sex pooled, *n* = 6) following single IV administration of Cytopeutics® hUC-MSCs in DRF and sub-chronic studies. Plasma cytokine levels including TNF-α, IL-6 and IL-10 were measured after (*a*) 14-d and (*b*) 90-d of observation. Bar graph represent the average concentration for each sample with different doses of Cytopeutics® hUC-MSCs (2 to 40 × 10^6^ cells/kg) or vehicle with vertical lines on each bar indicate standard error mean (SEM). *p* values were calculated using one-way ANOVA with Dunnett’s for multiple correction test (*p* < 0.05). The horizontal bars indicate pairwise comparison between vehicle with different doses.

Next, we determined the immunomodulatory action following a single IV administration of Cytopeutics® hUC-MSCs in healthy BALB/c mice by evaluating the changes in systemic plasma levels of the inflammatory cytokines TNF-α, IL-6 and IL-10 at different doses, followed by 14- (DRF study) and 90-d observation period (sub-chronic study) (Table 3). In DRF study (Fig. 4*a*, *b*), we observed that all Cytopeutics® hUC-MSCs-treated groups displayed a decreasing trend in plasma level of TNF-α and IL-6 in dose-dependent manner at 14 d after cell injection. Mice treated with 40 × 10^6^ cells/kg BW showed a significant reduction of TNF-α level when compared to the vehicle control group (0.86 ± 0.15 vs 3.10 ± 0.72 pg/mL, *p* = 0.034). Plasma levels of IL-6 were also significantly lower in mice injected with with Cytopeutics® hUC-MSCs in a dose-dependent manner when compared to control (30.10 ± 11.63 pg/mL). The reduction in IL-6 were noted with 5 × 10^6^ cells/kg BW (6.76 ± 5.07 pg/mL; *p* = 0.038), 20 × 10^6^ cells/kg BW (0.14 ± 0.00 pg/mL; *p* = 0.007) and 40 × 10^6^ cells/kg BW (2.90 ± 1.74 pg/mL; *p* = 0.014) × 10^6^ cells/kg BW. No significant difference was observed in IL-10 levels in plasma of the mice across the groups (Fig. 4*c*). Additionally, none of the measured cytokines were significantly different between the 15 × 10^6^ cells/kg BW and 40 × 10^6^ cells/kg BW groups compared to the vehicle control group in the sub-chronic toxicity study after 90 d of observation period.

**Table 3.** Cytokine levels in the plasma samples of mice (sex pooled, *n* = 6) following single IV administration of Cytopeutics® hUC-MSCs in DRF and sub-chronic studies

Type of study	14-d DRF dose toxicity study					90-d sub-chronic toxicity study		
Group	Vehicle control	2 × 10^6^ cells/kg BW	5 × 10^6^ cells/kg BW	20 × 10^6^ cells/kg BW	40 × 10^6^ cells/kg BW	Vehicle control	15 × 10^6^ cells/kg BW	40 × 10^6^ cells/kg BW
TNF-α	3.10 ± 0.72	2.16 ± 0.80	1.50 ± 0.64	1.31 ± 0.20	0.86 ± 0.15*	1.00 ± 0.20	0.73 ± 0.03	0.71 ± 0.02
IL-6	30.10 ± 11.63	12.77 ± 7.02	6.76 ± 5.07*	0.14 ± 0.00*	2.90 ± 1.74*	4.27 ± 2.82	3.07 ± 1.85	4.36 ± 1.90
IL-10	10.16 ± 2.88	6.12 ± 2.88	6.64 ± 2.17	4.63 ± 0.51	5.14 ± 0.00	3.10 ± 0.65	2.08 ± 0.00	2.59 ± 0.51

^*^Values were obtained from the DRF study (14 d) with single infusion (2 × 10^6^, 5 × 10^6^, 20 × 10^6^ and 40 × 10^6^ cells/kg BW) and 90-d sub-chronic study (15 × 10^6^ and 40 × 10^6^ cells/kg BW). Values are represented as mean ± SEM, where *p* < 0.05 compared to vehicle control. Plasma cytokine levels including TNF-α, IL-6 and IL-10 were measured at day 15 and day 91 of blood collection

### Gross pathology and histopathology analysis

**Figure 5.**
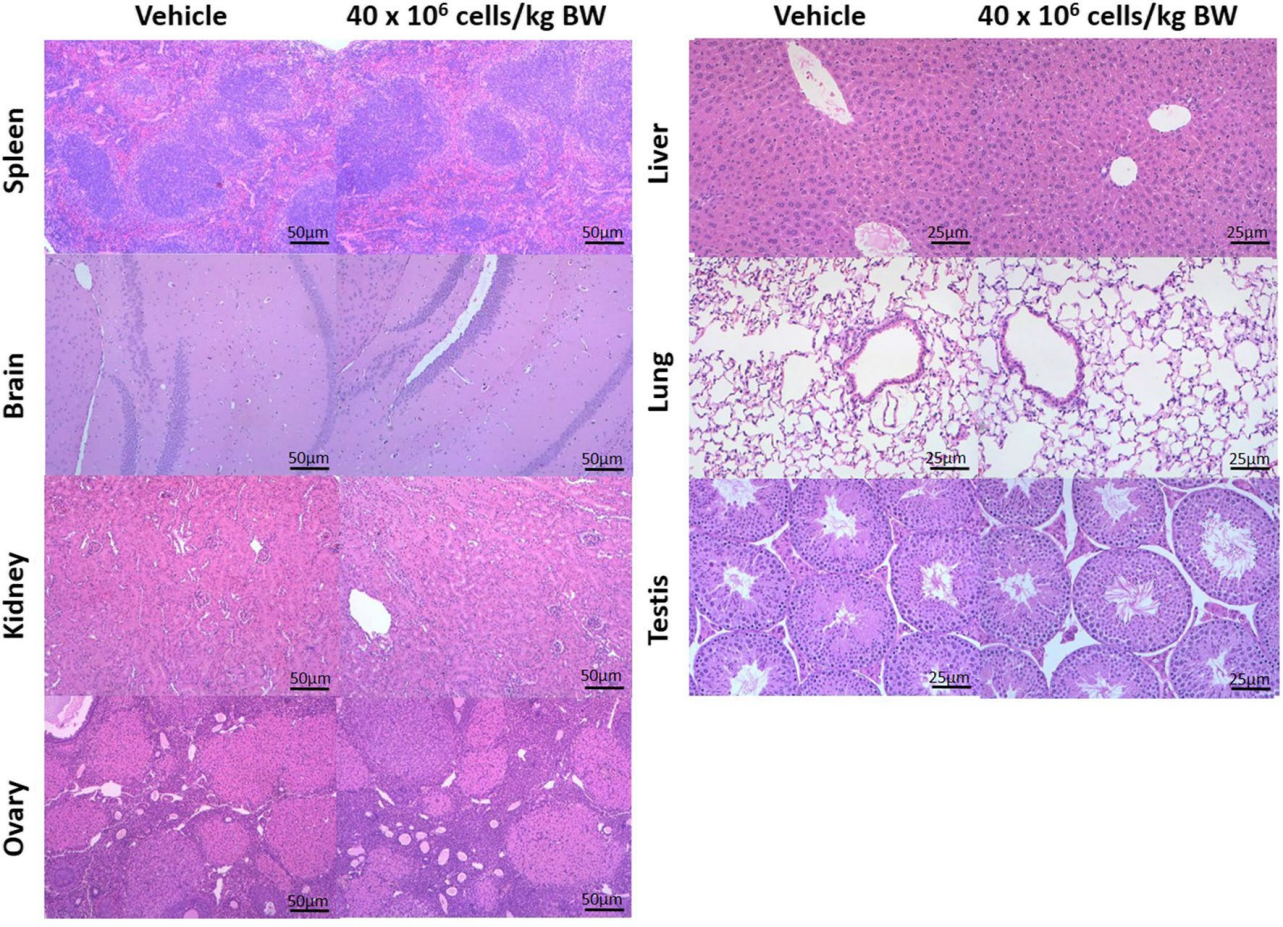
Representative H&E images of vehicle control and high dose of Cytopeutics® hUC-MSCs (40 × 10^6^ cells/kg BW) in selected organs. No microscopic changes induced by the hUC-MSCs in any of the organs examined from animals at 40 × 10^6^ cells/kg BW doses at the terminal sacrifice (day-91). Magnification × 100 (scale bar, 50 µm) and × 200 (scale bar, 25 µm).

In general, findings from all studies (DRF, 7-d and 28-d sub-acute, and 90-d sub-chronic studies) showed that there were no hUC-MSCs-related gross changes in the animals at terminal sacrifice except for a minimal 16% increment in the spleen weight which was observed in both 7-d and 28-d sub-acute studies using multiple dosing with 2 × 10^6^ cells/kg BW and 5 × 10^6^ cells/kg BW of Cytopeutics® hUC-MSCs respectively when compared to the control group. All other gross changes observed in animals of different groups were considered background/incidental finding commonly noted in laboratory mice of this age. As all mice were randomly distributed across the group based on different doses (from high dose to vehicle control group), any microscopic changes observed were considered incidental, spontaneous or age related which are common in laboratory mice with this age and strain (Fig. 5).

Histology analysis findings showed that there were no Cytopeutics® hUC-MSCs-related microscopic abnormalities observed in all organs or at the injection sites except for increased cellularity of white pulp in the spleen which was observed in the same 7-d and 28-d sub-acute studies. These changes were not seen in the sub-chronic study or the tumorigenicity study. In fact, there were no histopathological changes noted in any of the organs following single infusion of Cytopeutics® hUC-MSCs at 15 × 10^6^ cells/kg BW or 40 × 10^6^ cells/kg BW in the 90-d sub-chronic study.

### Tumorigenicity assessment in B-NDG SCID mice

**Figure 6.**
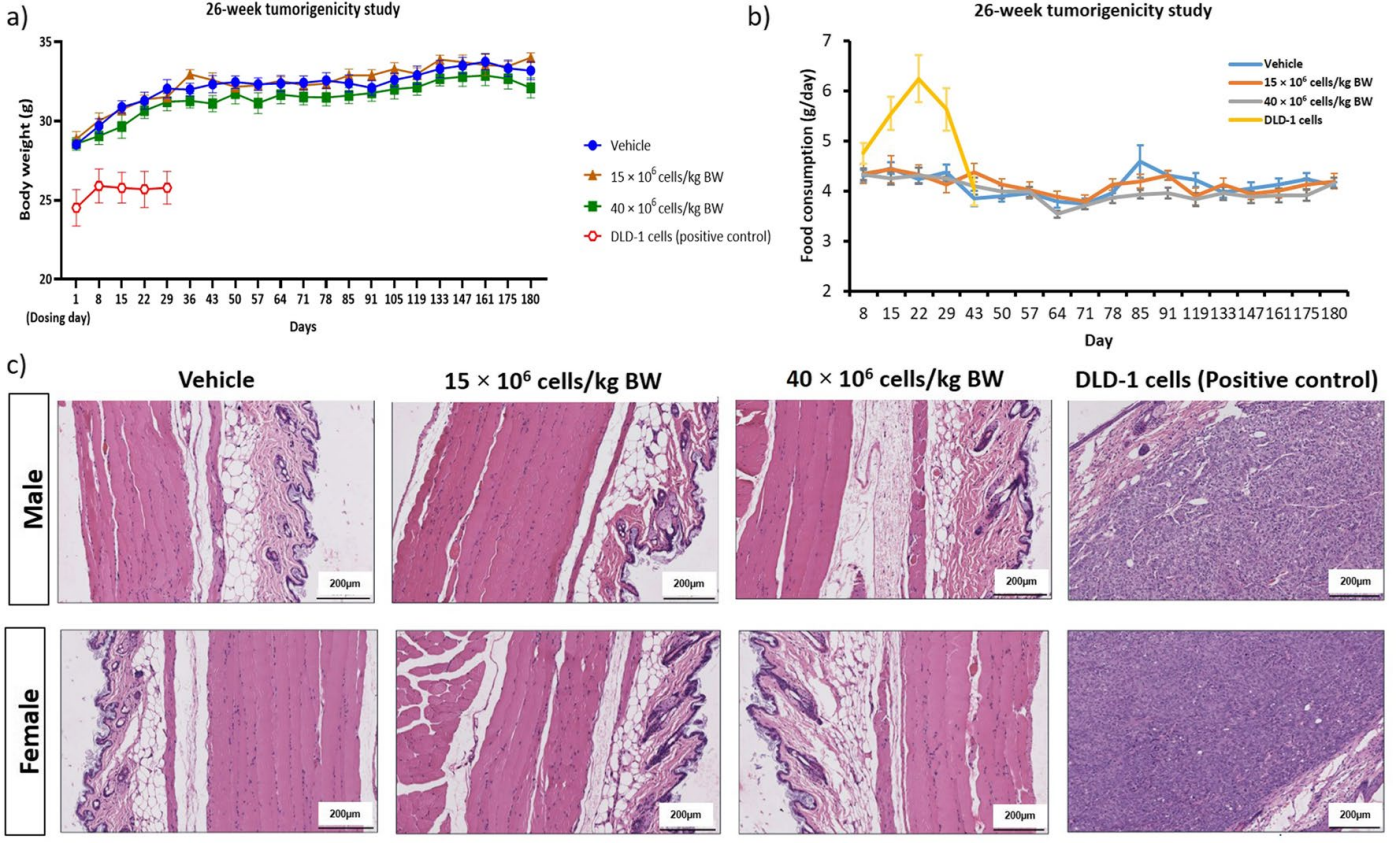
Analysis of body weight, food consumption, and histological section of B-NDG SCID mice in the 26-wk tumorigenicity study. Body weight (*a*) and food consumption (*b*) of the mice were identified to be consistent throughout the study period except for mice in the positive control (DLD-1 cells), where animals were euthanized after 32 d of observation period as the tumor size exceeded 10% of the animal’s weight. Histological examination with H&E staining at the site of the subcutaneous injection of the Cytopeutics® hUC-MSCs and DLD-1 cells in the skin of B-NDG SCID mice (*c*). Histological section at × 100 reveals normal morphological features of the skin between mice treated with vehicle, 15 × 10^6^ cells/kg BW, and 40 × 10^6^ cells/kg BW of Cytopeutics® hUC-MSCs. DLD-1 cells were injected to subcutaneous of B-NDG SCID mice to form tumors. Tumor growth is indicated by accumulation of neoplastic cells that extensively replaced the subcutaneous area of the injected site in the positive control group only. Magnification × 100, (scale bar, 200 µm).

There were no clinical signs of toxicity, body weight changes and abnormal food intake in all animals of vehicle control and at 15 × 10^6^ cells/kg BW and 40 × 10^6^ cells/kg BW of Cytopeutics® hUC-MSCs groups were observed, except for hair loss in one male at 15 × 10^6^ cells/kg BW and in three males at 40 × 10^6^ cells/kg BW until terminal sacrifice. Furthermore, no significant histopathological changes were noticed in 15 × 10^6^ cells/kg BW and 40 × 10^6^ cells/kg BW of Cytopeutics® hUC-MSCs, as well as the vehicle control group (Fig. 6). There was no evidence of tumor formation at the site of injection and in other organs of animals administered with Cytopeutics® hUC-MSCs at both doses at the terminal sacrifice (data not shown).

However, findings were different in the positive control group. All animals in the positive control group were humanely sacrificed on day-32 of study for ethical reasons as the tumor volume reached beyond 2000 mm^3^ in positive control group (DLD-1 cancer cells). During the observation periods, a decrease in body weight was observed in positive control mice, both males and females, from day-15 until terminal sacrifice (day-32) (Fig. 6*a*). A significant increased in food intake in male positive control mice was observed but considered transient as it declined over the observation period (Fig. 6*b*). Additionally, histological examination showed that the subcutaneous mass at the dorsolateral abdominal area observed during gross examination correlated microscopically as subcutaneous adenocarcinoma and attributed to DLD-1 cell lines administration (Fig. 6*c*). Also, there was hypertrophy of acinar cells along with decreased secretion in the granular duct of mandibular salivary glands and decreased secretion in parotid salivary glands in both sexes; atrophy of uterus in all female animals and decreased red pulp cellularity in spleen of a few female animals.

## Discussion

As far as we know, this present study is the first to demonstrate that IV administration of hUC-MSCs of up to 40 × 10^6^ cells/kg BW in a single dose did not cause clinical sign of toxicity and tumorigenicity in acute, sub-chronic and tumorigenicity studies in healthy BALB/c and immunocompromised B-NDG mice and is the maximum tolerable dose. Similar outcomes have been previously reported with lower doses of hUC-MSCs up to 10 × 10^6^ cells/kg BW in a single injection were also safe (Wang *et al* 2012; Kannaiyan *et al* 2017; Chan *et al* 2022; Xu *et al* 2022; Pan *et al* 2023).

Similarly, no tumor formation was observed in the skin injected with high dose Cytopeutics® hUC-MSCs or other vital organs of the treated mice in our study, except those in the positive control group. The immunocompromised mice model is known to be susceptible and is relatively sensitive for revealing the tumorigenic phenotype of a cell line and the changes observed in the positive control group were considered common and secondary to stress due to presence of tumor at the injection site and decreased body weight of the mice (Everds *et al* 2013).

In our study, multiple infusions of hUC-MSCs apparently minimally increased the splenic weight of the mice in both 7- and 28-d sub-acute studies. The enlargement of the spleen is characterised by an increased number of lymphocytes in the peri-arteriolar lymphoid sheath. There is evidence to suggest that this is a physiological response to IV MSCs which exert its immunomodulatory and therapeutic effects by migrating to secondary lymphoid organs (SLOs) such as the spleen which represents the main reservoir for immune cells (Acosta *et al* 2015; Luger *et al* 2017; Xue *et al* 2022). In fact, in the presence of acute injuries such as ischemic stroke, MSCs appear to largely persist in the spleen with only a small proportion homed to the target sites of injury upon IV administration (Wang *et al* 2019). Another study by Badner demonstrated that IV administration of hUC-MSCs significantly reduced spinal cord haemorrhage and increased IL-10 levels when the spleen is present, but the effect was diminished in rats with splenectomy (Badner *et al* 2018). While research on association of spleen and MSCs are limited, previous studies revealed that the spleen played a key role in ameliorating kidney injury by increasing Tregs and M2 macrophage proportion, mediated by MSCs (Hu *et al* 2013; Shimamura *et al* 2022). Despite the mild increase in splenic size, employing a multiple MSCs IV infusion strategy may still be safer in that it reduced the risk of thrombosis and possible pulmonary embolism seen with a single large infusion of 100 × 10^6^ cells/kg BW which represented the minimum lethal dose. Multiple infusions of MSCs may also be more effective to reduce chronic inflammation and sustain this effect compared to a single infusion. Multiple infusions of intravenously administered UC-MSCs alleviate insulin resistance in type 2 diabetes mice whereas inadequate number of infused-MSCs failed to ameliorate the insulin resistance in obese mice (Hao *et al* 2013; Nyamandi *et al* 2014). Furthermore, IL-10 levels, M2 macrophages and insulin sensitivity were elevated after multiple infusions of UC-MSC in epididymal adipose tissue of high fat diet–induced mice (Xue *et al* 2022).

The immunomodulatory properties of MSCs to regulate both innate and adaptive immune response in in vitro and in vivo are well-known (Moll *et al* 2015; Dabrowska *et al* 2021). Remarkably, we demonstrated that all mice treated with Cytopeutics® hUC-MSCs displayed a decreasing trend of TNF-α and IL-6 levels in the plasma in a dose-dependent manner. As all the mice were healthy, therefore our findings suggest that high doses of hUC-MSCs have the potential to counteract even subclinical pro-inflammatory states. The findings were in line with our previous work where TNF-α levels were reduced and the anti-inflammatory IL-1 receptor antagonist were elevated in a dose-dependent manner in healthy volunteers treated with hUC-MSCs (Chin *et al* 2020).

Our findings were also consistent with other studies that reported on MSCs infused in murine models which showed significant immunomodulatory effects by suppressing pro-inflammatory cytokines such as IFN-γ, IL-6, IL-8 and TNF-α (Liu *et al* 2018; Huang *et al* 2022). The role of TNF-α and IL-6 in regulating immune system is well-described in inflammatory-mediated disorders, autoimmune diseases or infection (Gabay 2006; Kany *et al* 2019). TNF-α is a fundamental cytokine in the inflammatory cascade and excessive activation of TNF-α signalling mediates apoptosis and inflammation (Webster & Vucic 2020). Meanwhile, IL-6 is considered to be unique due to its pleotropic activity as pro- or anti-inflammatory factor. The release of IL-6 is associated with activation and proliferation of lymphocytes, leukocyte recruitment and induction of an acute inflammatory response (Stenvinkel 2005). Attenuation of inflammation by MSCs has been well described in animal studies with inflammatory diseases including sepsis (Pedrazza *et al* 2017), type 2 diabetes (Sun *et al* 2017) and hepatic ischemia (Yao *et al* 2019).

Single administration of Cytopeutics® hUC-MSCs (15 × 10^6^ cells/kg BW and 40 × 10^6^ cells/kg BW) in the sub-chronic study did not show a significant difference of the levels of TNF-α, IL-6 and IL-10 levels in plasma of healthy mice. In fact, all the cytokine levels remained low (< 4 ρg/mL) after 90 d suggesting that the treatment did not induce any unwanted side effects or inflammation in long term. The findings were supported by previous work that demonstrated human BM-MSCs (10 × 10^6^ cells/mouse) injected in rodents did not change serum cytokine levels (IL-1β, TNF-α and IFN-γ) after 90 d of observation period (Rengasamy *et al* 2016). Similarly, Beggs and colleagues reported that intramuscular injection of MSCs at 5 × 10^6^ cells/kg BW in healthy baboons did not result in any change on the overall health and immune status of the animals after 12 wk (Beggs *et al* 2006). Indeed, low levels of the cytokines were associated with the regulation of homeostasis between cytokines network with the tissue microenvironment in the healthy mice, in the absence of injury and inflammation when the body is healthy. Therefore, the unaltered cytokines levels together with normal haematological and biochemical parameters of the mice have further confirmed the long-term safety of the Cytopeutics® hUC-MSCs up to the maximum tolerated dose of 40 × 10^6^ cells/kg BW in healthy mice.

## Conclusion

Systemic administration of allogeneic Cytopeutics® hUC-MSCs at the dose of up to 40 × 10^6^ cells/kg BW was safe and well tolerated in healthy BALB/c mice and B-NDG immunocompromised mice with no adverse reactions including allergic reactions. A dose-dependent immune-modulatory anti-inflammatory effect was observed with single IV administration of the cells between 2 and 40 × 10^6^ cells/kg BW. There was no sub-chronic toxicity observed up to 90 d nor with multiple infusions of Cytopeutics® hUC-MSCs at frequent intervals. Furthermore, Cytopeutics® hUC-MSCs were non-tumorigenic and could potentially act as immunomodulatory agent to further reduce sub-clinical inflammation. Due to promising outcomes of the present study, we are currently embarking on clinical trials of inflammatory-related diseases including GVHD, stroke, diabetes, heart disease and frailty to evaluate the efficacy of the Cytopeutics® hUC-MSCs.

## Data Availability

All data generated during this study conducted are included in this published article or are available from the corresponding author upon reasonable request.
